# Building Confidence, Diminishing Stress: A Clinical Incivility Management Initiative for Nursing Students

**DOI:** 10.3390/nursrep14030183

**Published:** 2024-09-19

**Authors:** Younglee Kim, Yeon Sook Kim, Henrietta Nwamu, Anne Lama

**Affiliations:** Department of Nursing, California State University San Bernardino, 5500 University Parkway, San Bernardino, CA 92407, USA; younglee.kim@csusb.edu (Y.K.); yeon.kim@csusb.edu (Y.S.K.); henrietta.nwamu@csusb.edu (H.N.)

**Keywords:** clinical incivility, intervention, nursing students, stress, self-efficacy

## Abstract

Objective: The aim of this study was to evaluate the effectiveness of an interactive program designed to reduce nursing students’ perceived stress and improve self-efficacy and readiness to professionally address incivility during clinical practice. Background: Incivility in clinical settings adversely impacts learners, educators, institutions, and healthcare systems, undermining safety and the teaching–learning process. Despite its increasing global prevalence, effective interventions remain largely unexplored. Methods: Our mixed-methods study, conducted from March to April 2024, involved senior baccalaureate pre-licensure nursing students (N = 35) from a California State University. The three-week, one-hour-per-week, interactive clinical incivility management program was developed through an extensive literature review. Pre- and post-intervention differences were assessed using a 10 min self-administered online survey that included the Uncivil Behavior in Clinical Nursing Education (UBCNE; 12 items), Perceived Stress Scale (PSS; 10 items), General Self-Efficacy Scale (GSE; 10 items), and a sample characteristics questionnaire (11 items). A one-hour face-to-face focus group (n = 11) then provided qualitative data on personal experiences of clinical incivility. Quantitative data were analyzed using SPSS version 27, while qualitative data were analyzed using Colaizzi’s method. Results: Clinical incivility prevalence was 71.4% (n = 25 out of 35). No statistically significant differences were found in UBCNE, PSS, and GSE scores between pre- and post-intervention. However, professional responses to clinical incivility significantly improved after the intervention (t = −12.907, *p* < 0.001). Four themes emerged from the qualitative data: (a) uncivil behaviors or language from nurses, (b) emotional discouragement and low self-confidence, (c) resource and personnel shortages at clinical sites for education, and (d) the necessity for interventions to manage clinical incivility. Conclusions: Nursing schools and clinical agencies should collaborate to establish monitoring systems, enhance communication, and implement evidence-based policies and interactive interventions to prevent and manage clinical incivility experienced by nursing students from clinical sites.

## 1. Introduction

Civility encompasses genuine respect and consideration, which are demonstrated through actions, the investment of time, and the language used to engage others. It promotes an environment that embraces differences while striving for common ground [1]. Conversely, incivility fosters an atmosphere of uncertainty, profoundly impacting both emotional and physical well-being [2]. In professional settings, incivility violates social norms and is categorized as deviant behavior [3,4]. Examples of uncivil behavior include bullying, intentionally ostracizing peers or subordinates, showing disregard for others, and using hostile actions or language [3]. These behaviors not only undermine interpersonal relationships but also erode trust and hinder collaboration within teams. They contribute to a negative work environment where individuals feel disrespected, which can lead to decreased job satisfaction and productivity [5]. Therefore, fostering a culture of civility is essential for promoting a supportive and effective workplace environment [1].

Incivility in healthcare has been documented to harm victims’ perceptions of self-worth and, in some cases, has resulted in violence [6]. In 2019, the American Nurses Association (ANA) [7] identified incivility as an issue affecting nursing at all levels, noting that bullying, incivility, and workplace violence consistently harm the healthcare system, team members, and patients. The ANA Code of Ethics for Nurses assigns nurses the responsibility of cultivating an ethically competent workplace that displays high levels of civility and states that students, as key stakeholders, must be provided with an environment of civility [8].

Pre-licensure (PL) undergraduate nursing students in Korea, China, the United States, and elsewhere often encounter rudeness during clinical practice, especially in hospitals; this troubling trend is worsening globally and threatens the culture of safety and the teaching-learning process in nursing education [9,10]. Clinical practice in nursing education is crucial for students as it fosters the development of practical skills, professional socialization, critical thinking, and communication proficiency, while exposing them to diverse patient care scenarios [11,12]. It provides hands-on experience where nursing students apply classroom knowledge into real healthcare settings, nurturing their competence and confidence essential for effective nursing practice. Additionally, clinical practice aids in preparing nursing students for licensure exams by fulfilling practical experience requirements set by licensing boards [13]. However, instances of uncivil behavior or inappropriate language can profoundly affect their academic outcomes, safety, and interpersonal relationships, potentially impeding learning during clinical practice [14].

Research on clinical incivility experienced by nursing students has been extensive in recent years, underscoring its severity and the urgent need for interventions to support nursing students [9,15]. Despite this, there remains a lack of appropriate educational programs or interventions to help nursing students prevent and manage incivility during their clinical practicum. Therefore, the objectives of our study were to (1) explore nursing students’ experiences with clinical incivility, (2) assess the effectiveness of an interactive program aimed at reducing perceived stress related to clinical incivility, and (3) enhance nursing students’ self-efficacy and readiness to professionally address incivility during their clinical practicum. The hypotheses were that, following participation in the interactive clinical incivility management program, senior nursing students in a pre-licensure BSN program would report (a) reduced levels of perceived stress, (b) increased general self-efficacy, and (c) enhanced preparedness to professionally respond to incivility during their clinical practicum.

## 2. Materials and Methods

### 2.1. Theoretical Conceptual Framework

In our research, we applied two theoretical models: Lazarus and Folkman’s cognitive theory of stress [16] and Bandura’s self-efficacy theory [17]. These models have been previously used to investigate the experiences of pre-licensure Bachelor of Science in Nursing (PL BSN) students during their clinical practicum [9]. Our study focused on two primary concepts from these theories: (i) perceived stress related to clinical incivility and (ii) self-efficacy. Lazarus and Folkman’s theory suggests that stress outcomes can be either positive or negative, depending on how individuals cope with stressors. These coping mechanisms are shaped by perceived threats and personal characteristics. Additionally, stressful environments can negatively affect the learning experience [18].

Bandura’s theory defines self-efficacy as a person’s confidence in their ability to perform tasks necessary to reach certain goals [17,19]. This concept involves believing in one’s capability to manage actions, emotions, and motivations to tackle challenges, accomplish objectives, and complete assignments [17,20]. Greater self-efficacy is associated with enhanced self-motivation, a deeper dedication to learning, and improved academic performance [21].

### 2.2. Study Design

Our study employed a mixed-methods approach, combining both quantitative and qualitative techniques. To gain a deeper understanding of study participants’ personal experiences of clinical incivility, a mixed-methods design was used, integrating both quantitative and qualitative data. This approach enhances the validity of research findings and addresses study limitations by using multiple data sources [22]. In the first phase, we conducted a quantitative analysis using a pre- and post-experimental design involving a 10 min self-administered online survey. This survey included the Uncivil Behavior in Clinical Nursing Education (UBCNE) with 12 items, the Perceived Stress Scale (PSS) with 10 items, the General Self- Efficacy Scale (GSE) with 10 items, and a questionnaire on sample characteristics with 11 items. The second phase featured a qualitative approach, comprising a one-hour in-person focus group aimed at collecting detailed personal experiences related to clinical incivility.

### 2.3. Ethical Considerations

Approval from the Institutional Review Board (IRB-FY2024-195) was secured from California State University on 29 February 2024. A total of 35 participants chose to attend the in-person clinical incivility management program after giving written informed consent. Out of these, 11 participants agreed to participate in an additional in-person focus group session after completing further consent forms. The remaining participants declined the focus group for personal reasons or due to a lack of interest.

### 2.4. Study Setting and Participants

To minimize sampling bias in our study, we selected a single cohort from one location and timeframe. We recruited senior nursing students from the PL BSN program at one California State University during the spring semester of 2024. The criteria for participation included being 18 years or older, proficient in English, and enrolled as a senior in the BSN program. Out of the recruited cohort, 35 nursing students voluntarily completed the quantitative survey, and their data were fully analyzed. This study required a minimum sample size of 34, determined by a power analysis with an alpha of 0.05, a power of 0.80, and a medium effect size of 0.50 (G*Power 3.1.9.7). [23]

### 2.5. Instruments

Quantitative data were gathered using a 10 min self-administered online survey featuring four questionnaires. These included the Uncivil Behavior in Clinical Nursing Education (UBCNE) to assess experiences with clinical incivility (12 items); the Perceived Stress Scale (PSS) for measuring stress levels (10 items); the General Self- Efficacy Scale (GSE) to evaluate perceived self-efficacy (10 items); and a sample characteristics questionnaire (10 items).

The UBCNE, developed by Anthony and Yastik [24], initially comprised 20 items designed to gauge nursing students’ experiences with clinical incivility. Subsequently, Anthony et al. [25] revised it to 12 items, organized into two subscales: Hostile/Mean/Dismissive Behaviors (H-M/dismissive; 7 items) and Exclusionary Behaviors (EXBEV; 5 items). Scores are based on a 5-point Likert scale (0 = never to 4 = very often), with total scores ranging from 0 to 48, where higher scores indicate greater experiences of incivility. In Anthony et al.’s study [25], the UBCNE had Cronbach’s alpha coefficients between 0.84 and 0.86. In our study, Cronbach’s alpha ranged from 0.84 to 0.88.

The Perceived Stress Scale (PSS), originally a 14-item self-administered questionnaire developed by Cohen et al. [26], was designed to measure perceived stress from various non-specific life situations over the past month. For our study, we utilized the 10-item version of this scale (PSS-10) as revised by Cohen and Williamson [27]. This version includes 10 items with responses rated on a 5-point Likert scale (0 = never to 4 = very often), with total scores ranging from 0 to 40. Higher scores reflect greater perceived stress. The PSS-10 had a Cronbach’s alpha coefficient of 0.82 in a recent study by Ashraf et al. [28] involving a Pakistani population. In our study, Cronbach’s alpha coefficient was 0.62.

We also employed the General Self-Efficacy Scale (GSE), created by Schwarzer and Jerusalem in 1995 [29]. This scale comprises 10 items, each rated on a 4-point Likert scale (1 = not at all true to 4 = exactly true). Total scores can range from 10 to 40, with higher scores indicating greater self-efficacy. Farnia et al. [30] reported Cronbach’s alpha coefficient of 0.94 for the GSE. In our study, Cronbach’s alpha coefficient was 0.89.

Our research team created a question to assess perceived readiness to handle incivility, based on a thorough literature review: “I feel prepared to respond professionally and appropriately when faced with uncivil behaviors in a clinical setting”. This item was rated on a 5-point Likert scale (1 = Strongly Disagree to 5 = Strongly Agree). To ensure the question’s clarity and readability, it was tested with five nursing students from a previous study by Kim et al. [9]. Additionally, the sample characteristics questionnaire consisted of 11 items covering demographics such as age, ethnicity, marital status, education, employment status, household income, religion, and experiences with clinical incivility.

Qualitative data were collected through an in-person focus group session using six open-ended questionnaires that were developed based on an extensive literature review and piloted with nursing students. The six questions for the focus group session were as follows:

(1) When you hear the word clinical incivility, what comes to your mind?
(2) How was your experience with nurses during your clinical practice?
(3) How did you react when you experienced incivility during your clinical practice?
(4) What do you think are common reasons for incivility during clinical practicum?
(5) How should clinical incivility be addressed in nursing schools or clinical agencies?
(6) Is there anything that you would like to add?

### 2.6. Procedure

The intervention in our study consisted of a concise, interactive program designed to manage clinical incivility, created by our research team following a review of both quantitative and qualitative studies on incivility management intervention written in English [6,31,32].

After completing the pre-intervention self-report online survey, a female nursing faculty member with a doctoral degree conducted three one-hour sessions per week with 35 participants in a classroom at California State University from March to April 2024. To ensure the successful execution of the intervention, this researcher had several meetings with team members to review educational materials and become familiar with the process and content. The post-intervention self-report online survey was administered at the conclusion of the third week’s session, and the study participants who completed it received a USD 10 gift card.

The three-week sessions were structured as follows:

Week 1 (60 min)

(1) Video presentation: Participants watched a video of incivility cases in healthcare settings (10 min).
(2) Small group discussion: Participants were subdivided into small groups of five or six students to discuss what they saw and felt about the video and then wrote a short discussion summary (20 min).
(3) Live researcher presentation on incivility impact and management: One research team member described studies on incivility definitions, incivility’s prevalence in nursing, examples of uncivil behaviors, and incivility’s effects on nursing education and practice (30 min).

Week 2 (60 min)

(1) Small group discussion: Participants shared individual experiences of incivility from their clinical sites in a small group by disclosing how they felt (30 min).
(2) Sharing with the entire group: Participants shared how they had responded to uncivil behaviors by writing on Google Suites collaborative digital whiteboard called Jam-board (30 min).

Week 3 (60 min)

(1) Live researcher presentation: One research team member lectured on strategies to professionally respond to incivility and incivility prevention (20 min).
(2) Practice formulating a professional response to clinical incivility using insights gained from the module: Participants drafted their responses to incidents of incivility, employing the Describe, Express, Specify and Consequences (DESC) Communication framework and Tool (https://www.aha.org/center/project-firstline/teamstepps-video-toolkit/desc, accessed on 11 April 2024) to structure their approach (20 min).
(3) Participants shared their written responses with the entire group (20 min).

On the same day after the intervention ended, two female members of the research team, both current university nursing faculty with doctoral degrees, conducted a debriefing focus group session with 11 participants. They had previously taught this cohort in required BSN courses at the same university and were experienced in nursing education research. For this focus group, they conducted a literature review to familiarize themselves with focus group methodology, discussed the process in detail, and completed training through team meetings to ensure effective facilitation. After explaining the purpose and process of the focus group and obtaining voluntary consent from the study participants in a different classroom at the university, the in-person session was conducted. The audio of the focus group session was recorded via the Zoom communication platform. During the 60 min session, the participants shared their experiences of incivility in the clinical practicum by responding to questions. Field notes taken during the focus group session were compiled by the two field researchers for subsequent discussion with the research members. After completing the focus group session, the study participants received another gift card valued at USD 10.

### 2.7. Quantitative Analysis

The coded data from all 35 participants in the Qualtrics surveys, with no missing pre- or post-intervention data, were used for quantitative analysis using SPSS Statistics software, version 27.0 (IBM Corp., New York, NY, USA).Descriptive statistical tests (e.g., frequency counts and distribution) were used to analyze participants’ characteristics, incivility experience, stress, self-efficacy levels, and self-reported preparedness to respond professionally to incivility. Our study employed a paired-samples *t*-test to evaluate changes before and after the intervention in measures of clinical incivility, perceived stress, and general self-efficacy. Pearson correlation analysis of the strength of relationships among the total scores on the UBCNE, the PSS, and the GSE was performed. To address hypotheses a, b, and c, a paired-samples *t*-test was used to determine whether the intervention of incivility management was effective.

### 2.8. Qualitative Analysis

During research team meetings, the field researchers’ notes from the focus group, participants’ feedback, and data saturation were reviewed and discussed prior to data analysis. The research team then employed thematic analysis [33] to analyze the qualitative data collected from the focus group. The participants’ voices were transcribed using the internet platform Zoom’s note-taking app called Zoom Notes (https://news.zoom.us/zoom-introduces-notes/, accessed on 11 April 2024). According to Colaizzi’s method for qualitative data analysis [34], all authors read the entire transcript of the focus group. Key statements were highlighted using the open-source, qualitative tool Taguette (https://www.taguette.org/about.html, accessed on 11 April 2024) and then analyzed and grouped into themes. The authors developed a narrative of participants’ experiences and summarized the findings into concise statements. Finally, they verified the findings with all authors to ensure accuracy.

## 3. Results

### 3.1. Phase One: Quantitative Data Analysis

Our study consisted of two phases: phase one involved quantitative data, while phase two involved qualitative data. In the quantitative phase, we analyzed survey data from a total of 35 senior nursing students who participated in pre- and post-intervention. The mean age of our sample was 23.29 years (SD = 4.11). The majority were Hispanic (n = 15, 42.9%), single (n = 30, 85.7%), and Catholic (n = 13, 37.1%). For more details on the sample characteristics, see Table 1. Of the 35 participants, 25 (71.4%) reported experiencing rude behaviors or words from nurses (n = 14, 40.0%), patients (n = 10, 28.5%), patients’ families (n = 3, 8.6%), and physicians (n = 1, 2.9%) from their clinical sites. In the results of paired-samples *t*-tests (Table 2), no statistically significant changes were found pre- and post-intervention of the UBCNE (t = −0.046, *p* = 0.963), PSS (t = 1.143, *p* = 0.261), and GSE (t = −0.762, *p* = 0.451); hypotheses a and b were not supported. However, the study participants’ scores for professional responses to clinical incivility significantly improved after the intervention (t = −12.907, *p* < 0.001); hypothesis c was supported. Pearson correlation coefficients among post-UBCNE, post-PSS, and post-GSE (Table 3) showed a significant positive correlation between the UBCNE and the PSS (r = 0.484, *p* = 0.003); this means that as nursing students’ feelings of incivility during their clinical practicum increase, their stress levels also tend to increase significantly.

### 3.2. Phase Two: Qualitative Data Analysis

In phase two, qualitative data from a single 60 min focus group (n = 11) were analyzed with the Taguette analysis program using Colaizzi’s method. Through the qualitative data analysis, four major themes were found: (a) uncivil behaviors or language from nurses, (b) emotional discouragement and low self-confidence, (c) resource and personnel shortages at clinical sites for education, and (d) necessity for interventions to manage clinical incivility.

#### 3.2.1. Uncivil Behaviors or Language from Nurses

The study participants reported experiencing both verbal and nonverbal abuse from nurses during their clinical practicum. The nurses sometimes appeared frustrated when answering students’ questions, doubted their knowledge and skills, and displayed displeasure, making them feel intimidated. Consequently, the study participants felt unwelcome and discouraged by the nurses in their clinical settings.

Participant # 2: Nurses usually roll their eyes or like once the students get in like assigned to them, they just don’t want to work with the students, or the nurses put the students to the side.

Participant # 5: In clinical, many nurses act really ignorant and rude towards student nurses.

Participant # 7: My nurse didn’t really say “Hi” or anything, completely ignoring me during the clinical.

#### 3.2.2. Emotional Discouragement and Low Self-Confidence

Due to clinical incivility, the students experienced emotional shock and a loss of confidence, making it difficult for them to continue with their clinical practice.

Participant # 7: I think it was a bad experience because the rude behavior and tone of voice of the nurses in the clinicals really lowered my self-confidence, and I think that memory stuck with me for a really long time.

Participant # 4: For me, my experiences with incivility created a snowball effect where bad experiences left me emotionally scarred and unable to acquire the skills, I needed to become more competent.

Participant # 8: It discourages me. I guess, it’s like knocking down my confidence and it just makes me feel like I am not good enough. My self-confidence was low, so it was difficult for me to continue the clinical.

Participant # 9: It hurts my emotions and really knocks my confidence down a lot.

#### 3.2.3. Resource and Personnel Shortages at Clinical Sites for Education

The study participants reported that clinical incivility seemed to arise from a lack of adequate resources and insufficient nursing staff to support student nurses’ practice at the clinical sites.

Participant # 5: I think that it’s a lot of the hospitals not preparing the nurses to have students.

Participant # 6: I just think there should be enough resources and nursing staff to teach us and prevent that incivility during clinicals.

Participant # 3: A lot of nursing students feel that incivility during their clinical practice. I think hospitals should have better educational environments… Sometimes the nurses are too busy to teach us, and it seems like they don’t really know how to educate us or how to support us.

#### 3.2.4. Necessity for Interventions to Manage Clinical Incivility

The study participants emphasized that clinical incivility is a serious issue affecting proper nursing education. Therefore, it is important for both hospitals and universities to develop and provide appropriate interventions and programs to help nurses and educators manage and prevent clinical incivility. Additionally, nursing students suggested that those preparing for their first clinicals should receive proper training on clinical incivility beforehand to handle it professionally.

Participant # 11: I think hospitals should implement training programs to supervise and educate nurses so that things like clinical incivility don’t happen.

Participant # 7: Both clinical instructors and nurses need to know how to identify, manage, and prevent incivility in clinical settings.

Participant # 10: The nursing students also need a training program to discuss what clinical incivility is, how to deal with it, and why it happens. I think it’s really important to understand and address these questions through discussion.

Participant # 1: Especially for nursing students starting their first clinical, it would be very helpful to have an educational program on clinical incivility before starting.

## 4. Discussion

In our study, the aim of investigating nursing students’ experience of clinical incivility was met. Consistent with previous research in the experience of incivility of nursing students in the clinical environment [9,35,36,37,38,39,40,41,42,43,44], 25 of the 35 participants (71.4%) reported experiencing incivility, with the majority of the behaviors coming from practicing nurses at their clinical sites (n = 14, 40.0%). As clinical education and hands-on practice is a mainstay requirement by boards of nursing and entities worldwide for licensure to practice, it is of no surprise following the history of nursing that the main perpetrator of incivility in the clinical environment are registered nurses [9,37,40,44,45]. The incidences of incivility shared by the participants in the focus group (n = 11) were experiences previously reported in traditional clinical education, which has been a mainstay of nursing education since Florence Nightingale’s model of apprenticeship training was established in formal nursing programs. Hospitals expect students to provide nursing care through on-the-job training as the focus of learning through observation and reporting [46]. However, the nursing student is just that, a student, learning how to be a nurse and, therefore, warrants an environment conducive to learning [47]. An environment conducive to learning requires civility. Civility is the foundation of an environment of learning and working, where all feel valued and where a sense of belonging is evident [48]. Nursing students are the future for a profession structured on the foundation of empathy, caring, and professionalism. Creating an environment of civility in nursing has been affirmed as essential by the ANA [7,8] in alignment with the Code of Ethics in Nursing, the International Council of Nurses Code of Ethics [49], and by the Tri-Council for Nursing [50], and the National League of Nursing [51] as crucial for fostering patient safety.

In the quantitative component of our mixed-methods study, we found that participants’ professional responses to clinical incivility significantly improved after the intervention (t = −12.907, *p* < 0.001), supporting hypothesis C: enhanced preparedness to professionally respond to incivility during their clinical practicum. After completing our interactive clinical incivility module, the data collected demonstrated PL BSN students in their senior year reported higher self-reported preparedness to professionally respond to incivility during the clinical practicum. This is supported by an e-learning module interventional program by Palumbo (2018) also utilizing Bandura’s conceptual framework utilizing recorded scenarios, slide presentations, and embedded pre-test/post-tests which 110 junior and senior nursing students completed. A significant increase via statistical analysis in results of the post-tests identified an increase in student self-efficacy in terms of both identifying and responding to incivility [52].

Additionally, a significant positive correlation was observed between UBCNE and PSS scores (r = 0.484, *p* = 0.003), indicating that, as feelings of incivility during clinical practicums increase, so do stress levels among nursing students. Similarly, a cross-sectional study by Bodys-Cupac et al. of 307 nursing students from Poland was completed to determine the relationship between perceived stress, using the PSS-10 by Cohen et al., self-efficacy, using the GSE by Schwarzer and Jerusalem, and coping strategies as it related to clinical practice. The data demonstrated that lower levels of self-efficacy correlated with higher levels of perceived stress (*p* < 0.0034) [53]. Unfortunately, our post-interactive program failed to statistically demonstrate lower perceived stress or higher general self-efficacy levels.

In the second phase, qualitative data collected from the focus group (n = 11) were analyzed thematically, revealing four main themes: descriptions of uncivil behaviors or language, primarily by practicing nurses; emotional discouragement and low self-confidence; resource and personnel shortages at clinical sites for education; and necessity for interventions to manage clinical incivility on all levels. Participants in Barbagallo’s study [36] shared their views on the clinical sites’ responsibility to take ownership of managing bullying issues, highlighting that such behaviors were commonly overlooked and inadequately addressed by management. The theme of uncivil behaviors or language from nurses can also be found in the literature worldwide, especially post-pandemic (Dias et al., El Ghaziri et al., Kennedy et al., Kim, Kim, et al., and Mammen et al. [9,42,54,55,56]). The overarching theme was shared by nine out of eleven participants in our focus group. Mean, disrespectful behavior on the part of the clinical nurses was corroborated by Ahn and Choi, who conducted a qualitative review of focus group interviews in South Korea with 32 senior nursing students, voicing a need to empower senior nursing students as well [57]. From subtle eye-rolling to outright ignoring nursing students’ questions, crucial for bridging nursing theory with clinical practice, the focus group highlighted these behaviors as disengaging and disconnecting, indicating a reluctance to interact with nursing staff and missing valuable learning opportunities. Similar findings were reported in a study by DiNatale [58] that followed nursing students into their initial roles as registered nurses post-graduation. However, despite these challenges, our focus group also included positive experiences. Participants shared stories of nurses who served as mentors in every aspect, although these accounts were primarily from two of the eleven participants.

The most sensitive theme was that of emotional discouragement and low self-confidence, with observations of frustration, disappointment, and tears. Shared experiences of feeling disempowered by the nurses’ perceived expectations of a level of mastery, which was, in their opinion, not being demonstrated, were reported; overhearing discussions of the nursing students being stupid created setbacks in the nursing students’ belief in their capacity to perform. Coping strategies to deal with the stress of clinical incivility, such as talking to a friend or family member, tearfully retreating to the unit bathroom to attempt to compose oneself to return, waiting until they left the clinical site to cry in private, as well as exercising, taking a walk, or listening to music, were shared. In an integrative review by Mellor et al. [59] focusing on psychosocial factors such as stress affecting new graduate registered nurses, it was found that the ability of clinical sites to provide workplace environments with enacted policies conducive to empowerment are variable due to incivility.

It is interesting to note that, when sharing themes of resource and personnel shortages at clinical sites, focus group members reflected with empathy on the challenges of clinical sites to accommodate nursing students. A qualitative meta-synthesis of nursing students’ use of reflective practice by Barbagallo [36] supported that improvement in practice is seen in the result of quality reflection. The qualitative piece of our study provided the participants with the opportunity to share their feelings on the lived experience of their future professional peers’ work challenges by looking through the eyes of the nurses they were assigned to. Reporting and empathizing with the challenges of mitigating staffing shortages, heavy patient assignments, and a lack of preparation to accommodate nursing students on units by the clinical sites demonstrates a competency in practice, while reflecting on knowledge of theory and practice leads to self-discovery. It is no secret that nursing practice has encountered challenges in the last several years.

This reflection resulted in the final theme of a need for action and change by all parties involved to mitigate incivility they may encounter through simulation and interventional programs prior to the start of their first clinical experience. The need for action and change by all parties involved included simulating real-life scenarios they may encounter but, more importantly, focusing on collaboration and focus on what our professional organizations have been stating in position statement on the topic. Clark’s *Competencies of Civility in Nursing and Healthcare* stresses the need for authentic civility, fostering inclusivity and belonging and thus enhancing individuals’ self-efficacy in achieving their goals—a cornerstone for the survival of nursing as a profession [48]. Mammen et al. [42], in their integrative literature review on the experiences of newly graduated nurses and workplace incivility in healthcare settings, noted that organizations’ failure to address bullying behaviors tacitly condoned incivility. The focus group echoed these findings, advocating for strategies outlined in the literature, such as shared governance and collaboration between nursing schools and clinical partners. They proposed early educational interventions to help nursing students recognize negative behaviors and respond effectively, along with advocating for clinical institutions to adopt a zero-tolerance policy towards workplace incivility [42]. As noted in Kim et al. (2023), earlier intervention, alternate formats, and/or additional content may have a greater impact on students’ stress and self-efficacy levels and other important outcomes.

To improve incivility in nursing education, the educational institution should create a code of conduct to encourage positive role models’ professionalism. In addition to taking personal responsibility to reduce actions as ongoing strategies to reduce incivility, Hudgins [60] suggested that faculty and students agree on solutions to reducing incivility by creating a code of conduct that defines acceptable and unacceptable behavior, role-modeling professionalism and civility, and taking personal responsibility and standing accountable for actions.

### Strengths and Limitations of This Study

The strengths of this study were the collection of both quantitative and qualitative data in research involving experiences of incivility in nursing students in clinical environments. It is important to acknowledge that perceptions of observable behaviors can vary widely depending on the situation and context in which they occur, supported by Stalter et al. [61]. Clark [48] highlights the significance of considering individual perceptions when reflecting on events involving verbal and nonverbal incivility, as it is the recipient of such behaviors who interprets the intent. Addressing limitations, our study focused exclusively on a single cohort of senior nursing students at a public university in the USA, which limits the generalizability of our findings to broader contexts of incivility experiences. Additionally, the participants in our focus group volunteered to take part, potentially introducing bias as their experiences with incivility may not be representative, especially given the small sample size and their relative inexperience with responding to incidents in clinical settings. Suggestions for future studies utilizing a personal interview to better understand nursing study participants’ expression of clinical incivility would be beneficial. We aimed to assess psychological changes, including stress levels and self-efficacy, among nursing students during a three-week interactive intervention. However, this timeframe may not have fully captured these changes, indicating the need for further research over an extended period. While our intervention focused on senior nursing students, our findings suggest that the interactive management approach to clinical incivility could be of more benefit in first-year nursing students preparing for clinical practice. Drawing on insights from Kim et al. [41], which indicated many nursing students feel prepared to handle clinical incivility based on their experiences, we recommend future intervention studies involving freshman nursing students before their clinical practicum begins.

## 5. Conclusions

Our exploration of nursing students’ experiences with clinical incivility further contributes to the plethora of shared outcomes and lived experiences of consistent themes of rude and offensive behaviors. What remains and has been identified throughout the literature is a gap in identifying strategies to promote civility and address incivility in nursing education and in clinical sites to prepare students for practice. Clark [48] emphasizes the urgent need for authentic civility with demonstrated core competencies of civility in nursing education, which these authors also support. Incorporating interactive learning programs and strategies into nursing curriculum, prior to the start of clinical practice and weaving it through courses as students’ progress, is vital to foster demonstration of civility in nursing practice. Although our hypothesis of reduced levels of perceived stress following participation in the interactive clinical incivility management program was not supported by the data, it did support the fact that nursing students are cognizant of the perpetuating presence of potential humiliation in their performance, which adds to the stress of wanting to learn from their clinical mentors.

Utilizing Bandura’s theory of self-efficacy quantitatively demonstrated that knowledge, practice, and reflection are power when it comes to emboldening nursing students to respond to the obvious lack of zero tolerance of rude and offensive behaviors, which remains a mainstay in clinical practice. Recommendations for promoting civility in nursing programs include orientation and simulation-based learning scenarios focused on reflections on incivility and professionalism and utilizing the DESC framework from the start of their program to empower nursing students to be prepared to respond to healthcare workers in the clinical environment. Ongoing communication with clinical sites is crucial to align nursing education with the promotion of authentic civility as a best practice, enforcing zero tolerance of uncivil behaviors.

## Figures and Tables

**Table 1:** Sample characteristics (N = 35).

Variable		Value
Age, M (SD)		23.29 (SD = 4.11)
Ethnicity *n* (%)	Hispanic or Latino	15 (42.9)
	Asian	10 (28.6)
	White	6 (17.1)
	Other	4 (11.4)
Marital Status, *n* (%)		
	Single/with no partner	30 (85.7)
	Married/living with partner	5 (14.3)
Education, *n* (%)		
	High school diploma	28 (80.0)
	Bachelor’s degree or higher	7 (20.0)
Employment, *n* (%)		
	Employed (full-time and part-time)	31 (88.6)
	Unemployed	4 (11.4)
Religion, *n* (%)		
	Catholic	13 (37.1)
	Christian	9 (25.7)
	Other	4 (11.7)
	Buddhist	1 (2.9)
	No religion	8 (22.6)
Annual household income, *n* (%)		
	Lesser than USD 20,000	5 (14.3)
	USD 20,000 to USD 49,999	6 (17.1)
	USD 50,000 to USD 99,999	15 (42.9)
	Over USD 100,000	9 (25.7)

Note: M = mean; SD = standard deviation.

**Table 2:** Comparison of pre- and post-intervention mean scores for study variables.

	Pre		Post					
Variable	M	SD	M	SD	*t(34)*	*p*	*r*	*Cohen’s d*
UBCNE	26.800	10.323	26.914	9.481	−0.046	0.963	−0.095	−0.008
PSS	32.542	4.175	31.285	5.533	1.143	0.261	0.123	0.193
GSE	30.171	4.598	31.000	4.419	−0.762	0.451	−0.017	−0.129

Note: Pre, before intervention; Post, after intervention; UBCNE, Uncivil Behavior in Clinical Nursing Education; PSS, Perceived Stress Scale; GSE, General Self-Efficacy Scale. M = mean; SD = standard deviation.

**Table 3:** Intercorrelations among the study variables (N = 35).

Variable	M	SD	Post-UBCNE	Post-PSS	Post-GSE
Post-UBCNE	26.914	9.481	1		
Post-PSS	31.285	5.533	0.484 **	1	
Post-GSE	31.000	4.419	−0.267	0.017	1

Note: Post, after intervention; UBCNE, Uncivil Behavior in Clinical Nursing Education; PSS, Perceived Stress Scale; GSE, General Self-Efficacy Scale. M = mean; SD = standard deviation. ** *p* < 0.01 (2-tailed).

## Data Availability

The data presented in this study are available from the corresponding author upon reasonable request.

## Public Involvement Statement

There was no public involvement in any aspect of this research.

## Guidelines and Standards Statement

This manuscript was drafted against the STROBE (Strengthening the Reporting of Observational Studies in Epidemiology) and COREQ (Consolidated criteria for Reporting Qualitative research) guidelines for mixed-methods research.
